# Lineage-Specific Sex-Biased Transcriptional Programs in Healthy Human Truncal Skin Revealed by Single-Cell Transcriptomics

**DOI:** 10.3390/genes17040415

**Published:** 2026-03-31

**Authors:** Yu Yang, Honghao Yu, Binbin Lai

**Affiliations:** 1 Institute of Medical Technology, Peking University Health Science Center, Beijing 100191, China; 2 Department of Dermatology and Venereology, Peking University First Hospital, Beijing 100034, China

**Keywords:** single-cell RNA sequencing, skin, truncal skin, sex-biased gene expression, sexual dimorphism

## Abstract

**Background/Objectives**: Sex differences influence skin physiology, immune regulation, and disease susceptibility, but the cellular organization of sex-biased transcriptional programs in healthy human skin remains incompletely defined. We aimed to define sex-associated differences in cellular composition and gene expression in healthy adult truncal skin at single-cell resolution. **Methods:** We constructed a sex-resolved single-cell transcriptomic atlas of healthy human truncal skin by integrating scRNA-seq data from 12 donors (5 males, 7 females). After quality control, 107,967 cells were classified into 14 major cell types. Sex-associated differences were assessed using donor-level pseudo-bulk analyses at both whole-skin and cell-type-resolved levels. **Results:** The cellular composition was conserved between sexes, with significant differences in mast cells and regulatory T cells. Whole-skin pseudo-bulk analysis identified distinct male-biased and female-biased transcriptional programs. Male-biased signals were linked to extracellular matrix organization and immune responses, while female-biased signals involved ion transport and neuromodulation. Cell-type-resolved analysis revealed that most sex-biased genes were lineage-specific, with minimal cross-lineage sharing. **Conclusions:** Sexual dimorphism in healthy human truncal skin is encoded through lineage-structured transcriptional regulation rather than broad compositional changes, providing a framework for understanding sex-biased skin homeostasis and disease susceptibility.

## 1. Introduction

Sex differences represent a fundamental axis of biological variation across human tissues, influencing physiology, immune regulation, metabolism, and disease susceptibility [1,2,3,4,5,6,7]. In skin, these differences have been linked to barrier properties, immune behavior, wound repair, and the incidence or severity of multiple dermatoses [8,9,10,11,12,13,14]. However, the cellular architecture through which sex-biased transcriptional programs are organized in healthy human skin remains incompletely resolved.

Previous transcriptomic studies have provided important insight but also have important limitations. Bulk RNA sequencing cannot resolve the cellular heterogeneity of skin and may therefore obscure lineage-specific transcriptional differences [4]. Existing single-cell studies of skin biology have more often focused on disease states, such as psoriasis or atopic dermatitis [12,13], or on highly specialized anatomical regions, including ear skin, hair follicles, and other site-restricted niches [14]. As a result, a systematic sex-resolved single-cell analysis of healthy human skin has remained lacking.

To address this gap, we performed a sex-resolved analysis of a single-cell RNA-sequencing (scRNA-seq) dataset of healthy adult truncal skin previously generated by our group and deposited in the Genome Sequence Archive (GSA accession: HRA007611), including 5 male and 7 female donors. Truncal skin provides an anatomically coherent reference site because it is moderately keratinized, experiences relatively balanced environmental exposure, and lacks the extreme specialization of palmoplantar, scalp, or genital skin [15]. This anatomical context enables a focused evaluation of intrinsic sex-associated differences while minimizing confounding effects related to marked site-specific physiology. After quality control, our dataset comprised 107,967 high-quality cells spanning the major epithelial, stromal, vascular, neural, and immune compartments of human skin.

Using this dataset, we asked whether sex-associated variation in healthy human skin is primarily reflected in differences in cellular composition, transcriptional regulation, or both. We show that male and female truncal skin share a largely conserved cellular framework, whereas sex-biased molecular programs are prominent and are organized predominantly in a lineage-structured manner.

## 2. Materials and Methods

### 2.1. Data Source and Study Design

The scRNA-seq data analyzed in this study were obtained from the Genome Sequence Archive (GSA) under accession number HRA007611. We retrieved raw sequencing files (FASTQ format) corresponding to scRNA-seq datasets of healthy adult human truncal skin. The dataset comprises 12 samples (7 females and 5 males), aged 23–48 years, with no reported dermatological conditions (Supplementary Table S1). Sample-level metadata, including donor age, anatomical site, and sequencing platform, were curated from the original data-generation project and used for downstream analyses. Beyond donor sex and chronological age, detailed endocrine metadata (e.g., menstrual cycle phase, oral contraceptive use, or perimenopausal status) were not available for this cohort. No donors had recorded hormone therapy in the available metadata. No donors had recorded hormone therapy in the available metadata. Ethical approval and informed consent were obtained in the original study. Only de-identified data were analyzed in the present work.

### 2.2. scRNA-Seq Data Analysis

Raw data processing: First, raw sequencing reads (FASTQ files) were processed using Cell Ranger [16] (v6.1.2). The gene-cell count matrices were generated using “cellranger count --include-introns”, and the human reference genome GRCh38-2020-A provided by 10x Genomics (https://cf.10xgenomics.com/supp/cell-exp/refdata-gex-GRCh38-2020-A.tar.gz, accessed on 18 June 2020) was employed. Initial preprocessing and quality control: Downstream preprocessing and quality control were performed using Scanpy [17] toolkit (v1.9.3) in Python v3.9. To mitigate contamination from ambient RNA, each sample was processed with DecontX [18] using default parameters, and the corrected count matrices were used for all subsequent analyses. Potential doublets were identified using Scrublet [19] (v0.2.3) independently for each sample, cells with scrublet scores above sample-specific thresholds corresponding to the right-tail peak of the simulated doublet distribution were removed. Then low-quality cells were excluded if they contained fewer than 1000 detected genes, more than 8000 detected genes, or more than 20% mitochondrial reads. After applying quality control, a total of 107,967 high-quality cells were retained across all donors. Integration: To correct for donor- and technical batch-associated variation, we used the scVI [20] implemented in scvi-tools [21] (v0.19.0). Specifically, the model was trained with the following parameters: n_latent = 50, n_layers = 3, dropout_rate = 0.1, and dispersion = “gene”. Batch covariates included sample_id as a categorical variable, and pct_counts_mt and total_counts as continuous variables. The resulting latent representation was visualized using UMAP. Identification of cell types: After integration, gene expression values were normalized in Scanpy (normalize_per_cell, counts_per_cell_after = 10,000) and log-transformed (log1p) for downstream clustering and annotation. Highly variable genes were identified using highly_variable_genes with batch_key = “sample_id” and flavor = “seurat_v3”. Unwanted variation associated with total_counts and percent_mito was regressed out, and the data were scaled with max_value = 10. PCA was computed for diagnostic purposes but was not used for neighborhood graph construction. For clustering and visualization, we constructed a k-nearest neighbor graph using the scVI latent representation (use_rep = “X_scVI”) with 20 neighbors, followed by Leiden clustering and UMAP visualization using sc.pp.neighbors(), sc.tl.leiden(), and sc.tl.umap(). Clusters were annotated using canonical marker genes from the literature and cross-validated against the original study annotations [22,23,24,25].

### 2.3. Statistical Assessment of Sex-Associated Differences in Cell-Type Proportions

To evaluate sex-associated differences in cellular composition, we quantified the proportion of each cell type within each donor as the fraction of cells assigned to that cell type relative to the total number of cells obtained from the same donor. Composition was assessed at both major cell-type and subtype levels. Only donor–cell type combinations containing at least 20 cells were included to avoid unstable estimates.

Male and female donors were compared using the Wilcoxon rank-sum test, which does not assume normality and is appropriate for small sample sizes. To complement significance testing, we also calculated Cliff’s delta as a nonparametric effect-size measure to quantify the magnitude and direction of differences between groups. All proportion analyses were performed at the donor level to ensure that biological rather than technical variation drove the comparisons. Because these analyses were intended to identify candidate shifts in cellular composition across a limited donor cohort, cell-type proportion comparisons were treated as exploratory and are reported using unadjusted two-sided *p* values, interpreted together with effect sizes unless otherwise specified. As a sensitivity analysis for donor-level composition findings, we modeled donor-level cell-type and subtype proportions using linear regression including age as a covariate (proportion ~ sex + age). Age-adjusted results are provided in Supplementary Table S2. In total, we performed 14 donor-level major cell-type proportion comparisons and 64 subtype-level proportion comparisons. Because these analyses are exploratory, *p* values are reported as unadjusted two-sided tests and interpreted together with effect sizes (Cliff’s delta).

### 2.4. Sex-Biased Differential Expression Analysis

To quantify sex-associated transcriptional differences while preserving donor-level biological replication, we applied a pseudo-bulk differential expression framework. For each cell type, raw UMI counts were aggregated by summing gene-level counts across all cells belonging to the same donor–cell type combination, yielding one pseudo-bulk profile per donor per cell type. A whole-skin pseudo-bulk matrix was generated in the same manner using all cells from each donor. For cell-type-resolved analyses, only donor–cell type combinations containing at least 20 cells were retained. Genes with fewer than 100 total counts across all samples were excluded. One major cell type, sweat gland cells (SGC), was excluded only from the cell-type–resolved pseudo-bulk sex-biased differential expression analysis because fewer than 20 cells were available in one or more donors within at least one sex group, precluding stable donor-level pseudo-bulk profiles. SGC were retained in atlas-level analyses, including cell-type annotation, UMAP visualization, and composition analyses, and their cells contributed to the donor-level whole-skin pseudo-bulk profiles. However, no SGC-specific cell-type–resolved sex-biased differential expression analysis was performed.

Pseudo-bulk matrices were normalized and adjusted for unwanted variation using the DaMiRseq [26] pipeline. Specifically, variance-stabilizing transformation (VST) [27] was applied via DaMiR.normalization function (minCounts = 10, fSample = 0.6) to normalize the gene expression. Low-quality pseudo-bulk samples were removed using DaMiR.sampleFilt function with th.corr = 0.7. we excluded 10 pseudo-bulk profiles (4 male-derived; 6 female-derived), originating mainly from Mast (*n* = 4), Schwann (*n* = 3), HFC (*n* = 2), and LC (*n* = 1). Excluded profiles are listed in Supplementary Table S3. Surrogate variable adjustment was performed using DaMiR.SVadjust function to mitigate residual technical and donor-specific effects. Differential expression (Female vs. Male) was performed using limma [28] (v3.52.4), modeling donor as the unit of replication. The significance threshold applied for Sex-biased genes (SBGs) was Benjamini–Hochberg FDR < 0.05 and |Log fold change| > log2(1.5).

Gene ontology enrichment analysis of SBGs was performed using clusterProfiler [29] (v4.4.4), testing Biological Process terms with Benjamini–Hochberg adjusted *p* value < 0.01 and minimum enrichment (GeneRatio/BgRatio) ≥ 1.5.

To explore potential disease relevance of baseline sex-biased programs, we compared SBGs identified in healthy truncal skin with GWAS Catalog risk-gene sets for skin diseases (psoriasis and atopic dermatitis). Disease-associated gene sets were obtained from the GWAS Catalog SNP–Phenotype Associations (2025 release) gene set library (distributed by Harmonizome). Overlaps between each SBG list (whole-skin and cell-type-resolved; all SBGs, female-up, and male-up subsets) and each disease risk-gene set were assessed using one-sided Fisher’s exact tests (alternative = “greater”). For each comparison, the background universe was defined as the set of genes tested in the corresponding SBG analysis (i.e., the genes present in the respective pseudo-bulk DE table after filtering). *p* values were adjusted for multiple testing across all overlap comparisons using the Benjamini–Hochberg procedure. Results are provided in Supplementary Table S4.

### 2.5. Cross Cell-Type Sharing Analysis of Sex-Biased Genes

To quantify the extent to which sex-biased genes were conserved across skin cell lineages, we performed a cross-cell-type SBG sharing analysis. For each cell type, female-upregulated and male-upregulated SBGs identified by the pseudo-bulk limma workflow were extracted separately. Binary presence–absence matrices (genes × cell types) were then constructed for female-upregulated and male-upregulated genes, where each entry was assigned a value of 1 if the gene was significantly upregulated in that cell type (FDR < 0.05 and |log2FC| > log2(1.5)) and 0 otherwise. Shared SBGs were summarized by counting genes present in at least n cell types (n = 1–14). Two types of visualization were generated: (i) bar plots showing the number of SBGs shared across different numbers of cell types, and (ii) binary heatmaps for genes detected in at least three cell types, highlighting recurrent lineage-spanning signatures. These analyses were performed in R using base functions and ComplexHeatmap [30] (v2.15.4) package.

### 2.6. Statistical Analysis

All statistical analyses were performed using R (v4.2.1) and Python (v3.9). Unless otherwise stated, all statistical tests were two-sided. Multiple testing was controlled using the Benjamini–Hochberg procedure, and FDR < 0.05 was considered statistically significant. Data visualization was performed using ggplot2 in R and standard Python plotting libraries.

## 3. Results

### 3.1. A Sex-Resolved Single-Cell Atlas of Healthy Human Truncal Skin Reveals Selective Male-Biased Immune Enrichment

**Figure 1:**
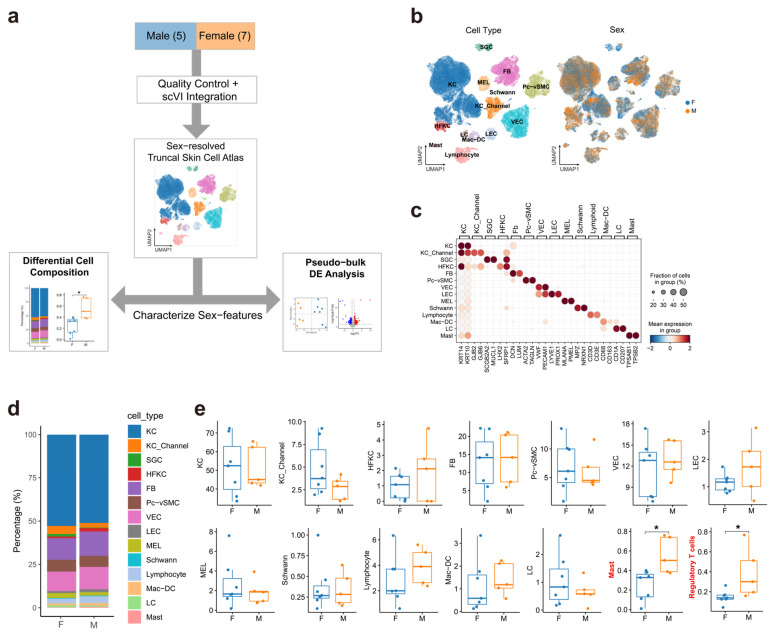
Study design and sex-associated cellular composition in healthy truncal skin. (**a**) Schematic overview of the study design and analytical workflow. (**b**) UMAP visualization of the batch-corrected dataset, colored by annotated cell type (left) and sex (right). Major cell populations included keratinocytes (KC), sweat gland cells (SGC), hair follicle keratinocytes (HFKC), melanocytes (MEL), Schwann cells, fibroblasts (FB), pericyte/vascular smooth muscle cells (Pc−vSMC), vascular endothelial cells (VEC), lymphatic endothelial cells (LEC), and multiple immune cell populations, including lymphocytes, macrophages/monocytes/dendritic cells (Mac−DC), Langerhans cells (LC), and mast cells. Within the keratinocyte compartment, we annotated a keratinocyte subpopulation enriched for ion-transport/gap-junction features (KC_Channel) [25]. (**c**) Dot plot showing canonical marker gene expression across major cell types. (**d**) Stacked bar plots showing cell-type proportions in each donor, grouped by sex. (**e**) Box plots showing donor-level proportions of major cell types stratified by sex. *p* values were calculated using the two-sided Wilcoxon rank-sum test, * *p* < 0.05.

We integrated and analyzed scRNA-seq data from 12 healthy adult human truncal skin donors (5 males and 7 females; aged 23–48 years; Figure 1a, Supplementary Table S1). After stringent quality control, 107,967 high-quality cells were retained for downstream analyses. Unsupervised clustering combined with canonical marker gene expression identified 14 major cell types (Figure 1b,c), providing the cellular framework for subsequent sex-stratified analyses of healthy human truncal skin.

Analysis of relative cell abundance across the 14 major cell lineages showed that the overall cellular composition of adult truncal skin was largely stable between sexes. No significant sex-associated differences were detected in 13 of the 14 major cell types (Figure 1d), indicating that sexual dimorphism in healthy truncal skin may not be primarily driven by global shifts in major cell-type proportions. Mast cells were a clear exception, showing a consistent and significant enrichment in male donors (*p* = 0.023, Wilcoxon test; Figure 1e), with a large effect size (Cliff’s delta = −0.829), consistent with a male-biased increase across the cohort. This observation is notable because previous studies of normal human skin did not report a significant sex difference in mast cell abundance [31], suggesting that the male-biased enrichment observed here may reflect anatomical site-specific effects or differences in analytical resolution. We then examined cellular composition at subtype resolution and identified regulatory T cells (Tregs) as a second male-biased population. Although no significant sex difference was detected for the corresponding major lymphocyte lineage, Tregs showed a significantly higher proportion in male truncal skin (*p* = 0.035, Wilcoxon test; Figure 1e), with a substantial effect size (Cliff’s delta = −0.771). This finding is consistent with prior evidence for sex-associated differences in Treg frequency and function [32], although comparable evidence in normal human skin remains limited.

Together, these results suggest a selective male-biased immune profile in healthy truncal skin, characterized by increased mast cells at the major cell-type level and increased Tregs at the subtype level, while the overall tissue composition remains largely conserved between sexes.

### 3.2. Whole-Skin Transcriptomes Reveal Sex-Biased Functional Programs in Healthy Truncal Skin

**Figure 2:**
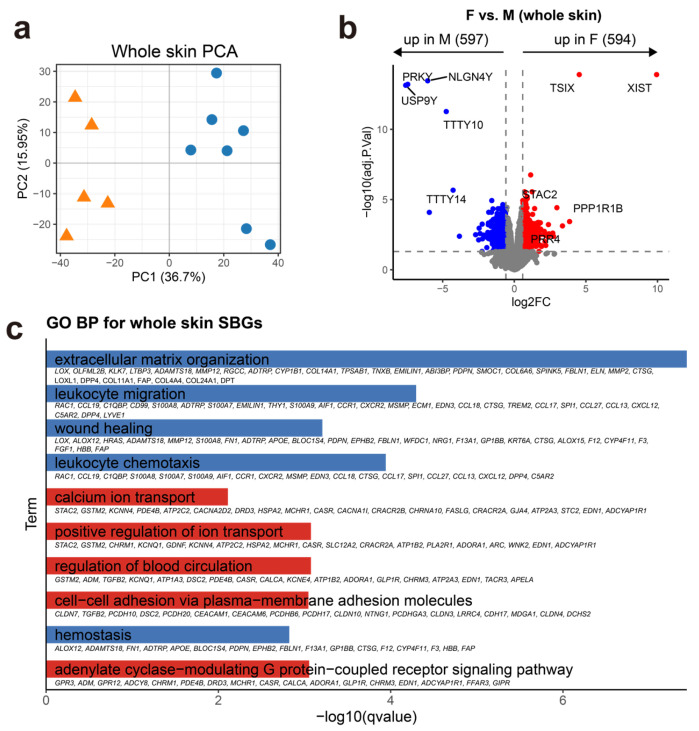
Whole-skin sex-biased transcriptional programs in healthy truncal skin. (**a**) PCA of donor-level whole-skin pseudo-bulk transcriptomes, showing sex-associated separation in transcriptomic space. (**b**) Volcano plot of sex-biased genes (SBGs) identified at the whole-skin level. Significantly differentially expressed genes (adjusted *p* < 0.05 and |log2FC| > 0.58) are highlighted in red (female-biased) or blue (male-biased). (**c**) Bar plot showing enriched Gene Ontology Biological Process (GOBP) terms for whole-skin SBGs.

Given the limited sex-associated differences in major cell-type composition, we next asked whether molecular dimorphism could be detected at the transcriptome level. Donor-level pseudo-bulk profiling revealed sex-associated separation in whole-skin transcriptomic space (Figure 2a). Consistent with this separation, we identified a moderate set of sex-biased genes (SBGs) (Figure 2b; Supplementary Table S5), suggesting that sex-associated transcriptional differences are detectable in healthy adult truncal skin even in the absence of broad compositional shifts. As expected, canonical sex-chromosome markers were among the most strongly biased signals, including XIST/TSIX in females and UTY/USP9Y/TTTY family genes in males (Figure 2b). Functional enrichment analysis showed that female-upregulated SBGs(F-SBGs) were associated with ion transport regulation, G protein-coupled receptor signaling, neuromodulatory activity, and cell–cell junction organization, suggesting sex-biased differences in epithelial and vascular signaling as well as regulatory processes. In contrast, male-upregulated SBGs(M-SBGs) were enriched for extracellular matrix organization, leukocyte migration, chemokine signaling, coagulation, and wound-associated pathways, suggesting stronger coupling to stromal remodeling and immune trafficking at baseline (Figure 2c).

These findings suggest that sex-biased transcriptional variation in healthy truncal skin is organized into distinct functional programs rather than reflecting a uniform shift in gene expression. In particular, male-biased whole-skin signals preferentially mapped to stromal remodeling and inflammatory trafficking pathways, whereas female-biased signals were enriched for signaling- and regulation-associated processes.

### 3.3. Tissue-Level Sex-Biased Transcriptional Signals Are Organized by Lineage-Specific Programs with Limited Cross-Lineage Sharing

**Figure 3:**
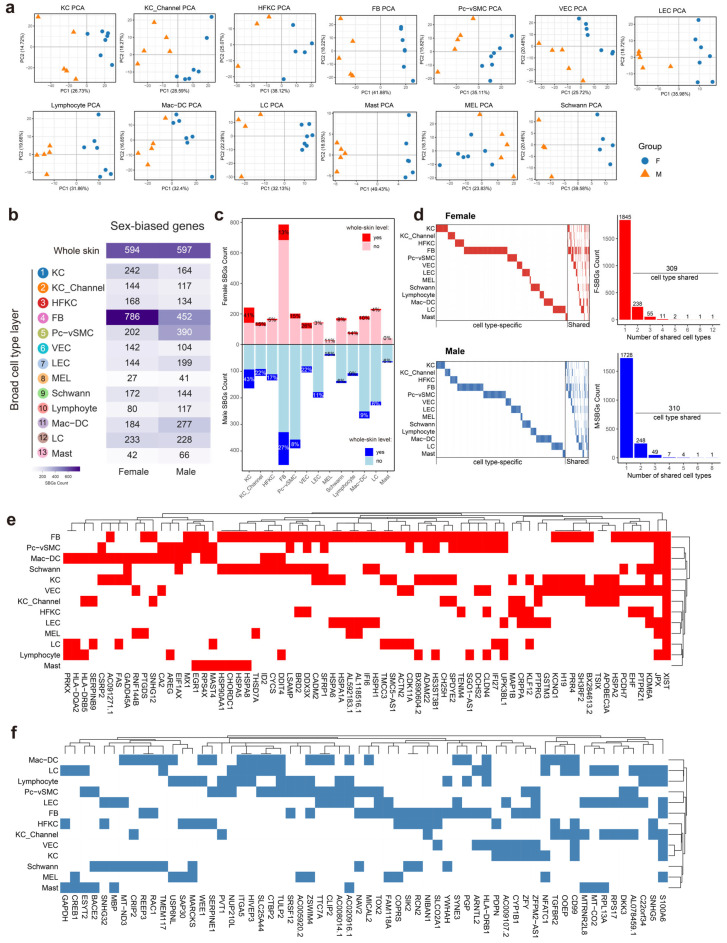
Lineage-specific sex-biased transcriptional programs in healthy truncal skin. (**a**) PCA of donor-level pseudo-bulk transcriptomes for each major cell type, showing varying degrees of sex-associated separation across lineages. (**b**) Heatmaps showing the numbers of female-biased (left) and male-biased (right) SBGs detected in each major cell type. (**c**) Stacked bar plots showing the proportion of female-biased SBGs (top) or male-biased SBGs (bottom) in each cell type that were also detected at the whole-skin level (highlighted in saturated colors). (**d**) Distribution of SBG sharing across cell types. Left, binary presence–absence matrices showing the distribution of female-biased (top) and male-biased (bottom) SBGs across major cell types. Right, bar plots showing the numbers of SBGs detected in exactly n cell types. (**e**,**f**) Binary presence–absence heatmaps showing lineage-sharing patterns of female-biased (**e**) and male-biased (**f**) SBGs detected in at least three cell types.

To define the cellular architecture underlying whole-skin sex-biased transcriptional signals, we examined donor-level pseudo-bulk transcriptomes across major skin cell types. PCA at cell-type resolution revealed varying degrees of sex-related separation across lineages (Figure 3a), indicating that transcriptional dimorphism is unevenly distributed across epithelial, stromal, vascular, neural, and immune compartments rather than reflecting a uniform tissue-wide shift. Consistent with this lineage dependence, the number of SBGs varied markedly across the major cell types (Figure 3b, Supplementary Table S6), with both F-SBGs and M-SBGs contributing to these patterns. This heterogeneity indicates that sex-associated transcriptional regulation in healthy truncal skin is predominantly cell-type specific, with distinct lineages exhibiting different magnitudes of sex bias. We next quantified how whole-skin SBGs relate to lineage-resolved transcriptional changes. Across most cell types, only a small fraction of lineage SBGs (3–27%) was detectable in whole-skin profiles (Figure 3c), indicating that tissue-level analysis captures only a subset of sex-biased variation present at cellular resolution. Among the major lineages, keratinocytes and fibroblasts contributed the largest absolute number of whole-skin SBGs (Figure 3c), consistent with their dominant contribution to the aggregate skin transcriptome.

Finally, we examined whether sex-biased transcriptional programs were broadly conserved across lineages. For both F-SBGs and M-SBGs, most SBGs were restricted to a single cell type (1845 F-SBGs and 1728 M-SBGs; Figure 3d), reinforcing strong lineage specificity. A smaller subset of SBGs was shared across multiple cell types, indicating limited but reproducible cross-lineage modules. Binary heatmaps of genes detected in at least three cell types showed that these shared signatures spanned epithelial, stromal, and immune compartments rather than being confined to a single lineage (Figure 3e,f).

Together, these results show that sex-biased transcriptional variation in healthy truncal skin is hierarchically organized: tissue-level signals arise primarily from strongly lineage-specific programs, with only limited cross-lineage shared modules.

## 4. Discussion

In this study, we constructed a sex-resolved single-cell atlas of healthy adult human truncal skin and used it to define sex-associated differences in cellular composition and transcriptional programs across skin lineages. Our analyses indicate that sexual dimorphism in healthy truncal skin is not characterized by broad shifts in major cell-type abundance, but rather by selective immune enrichment and widespread transcriptional divergence. Most major cell types were present at comparable proportions in males and females, whereas mast cells and Tregs showed male-biased enrichment. At the transcriptomic level, male and female skin displayed distinct whole-skin functional programs, and these tissue-level signals were primarily organized through lineage-specific transcriptional programs with limited cross-lineage sharing. Together, these findings suggest a model in which sex-biased skin homeostasis is encoded predominantly at the molecular level and distributed hierarchically across cell types rather than through large-scale remodeling of tissue composition.

A central observation of this study is the relative stability of cellular composition between sexes in healthy truncal skin. This finding suggests that male and female skin share a broadly conserved cellular scaffold under homeostatic conditions, despite well-recognized sex differences in skin physiology and disease susceptibility [14,32,33,34]. Such a pattern is important because it implies that sex-biased skin phenotypes may arise less from major differences in which cell populations are present and more from differences in how shared cell types are transcriptionally regulated. This distinction may be particularly relevant for understanding sex-biased dermatologic conditions in which inflammatory responsiveness, tissue remodeling, or barrier-associated signaling differ between sexes without overt changes in tissue architecture.

Within this generally conserved cellular framework, we identified two notable immune exceptions. Mast cells were enriched in male truncal skin at the major cell-type level, whereas Tregs showed male-biased enrichment at subtype resolution. The mast cell result is particularly notable because previous studies of normal human skin did not consistently identify a stable sex difference in mast cell abundance [31,35], suggesting that the signal observed here may reflect anatomical site-specific effects or differences in resolution between single-cell and conventional histomorphometric analyses. Increased Tregs in male skin are more consistent with broader immunologic evidence [32,36,37,38] suggesting sex-associated differences in Treg frequency and suppressive function, although comparable evidence within normal human skin remains limited [39,40]. Together, these findings suggest a selectively male-biased immune microenvironment in healthy truncal skin and indicate that sex-dependent differences in immune tone may already be present under non-diseased conditions. In this context, it is worth noting that sex-differential immune set points reported in mouse skin are not always directionally concordant with human observations, and may be shaped by species-, site-, and readout-dependent factors.

A recent mouse study by Chi et al. proposed an androgen–ILC2–DC axis underlying sex-differential skin immunity and reported higher accumulation of T cells (including Tregs) and a denser/activated DC network in females [14]. While this direction appears different from our observation of a male-enriched Treg proportion in adult human truncal skin, the two findings are not necessarily contradictory. Differences in species (mouse versus human), anatomical site and tissue context, and outcome measures (absolute accumulation/density and functional activation in mice versus donor-level cellular proportions derived from dissociated human biopsies) could all contribute to divergent apparent directions. Notably, evidence in healthy human skin does not uniformly support higher female Treg abundance, with prior studies reporting either no significant sex difference [41] or higher Treg numbers in males [42] depending on assay and readout. In addition, although ILC2 are present in human skin, they can be rare in steady state and may be under-sampled in whole-skin scRNA-seq, precluding a direct test of an analogous androgen–ILC2–DC axis in the present dataset. Taken together, these considerations motivate targeted validation in independent human cohorts using immunophenotyping and spatial assays, and they provide a mechanistic context for interpreting the lineage-resolved transcriptional programs described below.

At the whole-skin transcriptome level, we found that male and female truncal skin were distinguished by different functional programs rather than by a single uniform expression shift. Male-biased signals were associated with extracellular matrix organization, leukocyte migration, chemokine signaling, coagulation, and wound-associated pathways, whereas female-biased signals were enriched for ion transport, G protein-coupled receptor signaling, neuromodulatory activity, and cell–cell junction organization. These patterns suggest that healthy male and female skin may maintain tissue homeostasis through partially distinct baseline strategies, with male skin showing stronger coupling to stromal remodeling and inflammatory trafficking and female skin showing relatively stronger enrichment of signaling- and regulation-associated processes [14,43]. Importantly, these differences were evident in normal, non-lesional skin, indicating that sex-associated functional divergence is embedded within the baseline transcriptomic architecture of healthy tissue.

A major contribution of this work is the demonstration that tissue-level sex-biased programs are largely built from strongly lineage-specific transcriptional changes. The degree of sex-related separation varied across cell types, the number of sex-biased genes differed markedly by lineage, and only a minority of lineage-level sex-biased genes was detectable at the whole-skin level. In addition, most sex-biased genes were restricted to a single cell type, with only a limited subset shared across multiple lineages. These findings argue against a model in which sexual dimorphism in skin is driven by a common tissue-wide transcriptional program. Instead, our data support a lineage-partitioned architecture in which epithelial, stromal, vascular, neural, and immune compartments each contribute distinct components to the aggregate sex-biased phenotype. This framework may help explain why bulk tissue analyses often underestimate biologically meaningful sex differences in complex tissues.

To provide an initial genetics-based link between baseline sex-biased programs and disease relevance, we compared our SBGs with GWAS Catalog risk-gene sets for psoriasis and atopic dermatitis (Supplementary Table S4). Notably, male-biased fibroblast SBGs showed significant enrichment for atopic dermatitis risk genes (odds ratio = 6.76, overlap = 7 genes, BH-FDR = 0.0156), with overlapping genes including HLA-DQA1, HLA-DRB1, IL6R, LRRC32, OVOL1, TNFRSF6B, and TNXB. In contrast, overlaps for most other lineages were nominal and did not remain significant after multiple-testing correction. Although this analysis is exploratory and does not replace direct case–control comparisons in patient cohorts, it suggests that a subset of baseline sex-biased stromal programs may intersect with disease-relevant genetic architectures, motivating future validation in independent cohorts and disease contexts.

These observations may also provide a useful reference for interpreting skin diseases that show sex-biased incidence, severity, tissue behavior, or treatment responses [33]. If sex-biased transcriptional programs are already embedded in healthy skin and distributed across specific cellular compartments, then disease-associated sex differences may emerge through differential amplification of pre-existing lineage-restricted programs rather than through entirely disease-specific mechanisms. In this context, the lineage-structured organization of sex bias described here may be relevant to inflammatory skin disease, wound repair, fibrosis, and cutaneous tumors that exhibit sex-dependent clinical patterns.

Several limitations should be acknowledged. First, the cohort size was modest, which may limit power to detect more subtle compositional or transcriptional differences, particularly in rare cell populations. Second, this study was restricted to healthy adult truncal skin, and the extent to which these findings generalize to other anatomical sites, age groups, or hormonal states remains uncertain. Although no hormone therapy was recorded, detailed endocrine metadata (e.g., menstrual cycle phase or contraceptive use) were unavailable; therefore, immune-composition findings (mast cells and Tregs) should be interpreted as exploratory and warrant validation in independent cohorts with endocrine annotation and orthogonal immunophenotyping/spatial assays. Third, our conclusions are based on transcriptomic inference and do not directly establish functional differences in immune activity, tissue remodeling, or barrier regulation. Finally, although donor-level pseudo-bulk analysis improves robustness, residual inter-individual variation and unmeasured clinical factors may still contribute to some observed sex-associated signals.

## 5. Conclusions

This study provides a sex-resolved single-cell reference for healthy human truncal skin and shows that sex-associated molecular divergence is organized primarily through lineage-structured gene expression programs rather than broad shifts in tissue composition. These results offer a baseline framework for future studies of sex-biased skin homeostasis, immune regulation, and disease susceptibility.

## Data Availability

The raw scRNA-seq data analyzed in this study are available in the Genome Sequence Archive (GSA) under accession number HRA007611. Processed single-cell expression matrices, the integrated .h5ad object, and donor metadata have been deposited in Figshare and are publicly available at: https://doi.org/10.6084/m9.figshare.30715472. All original code has been deposited at GitHub and is publicly available at: https://github.com/yuybio/skin-sex (accessed on 7 March 2026).

## Supplementary Materials

The following supporting information can be downloaded at: https://www.mdpi.com/article/10.3390/genes17040415/s1, Table S1: Excel file containing sample information used in this study; Table S2: Age-adjusted sensitivity analysis of donor-level cell-type and subtype proportions using linear models (proportion ~ sex + age); Table S3: Pseudo-bulk profiles removed by DaMiR.sampleFilt (th.corr = 0.7), including donor ID, cell type, and sex; Table S4: Over-representation analysis of overlaps between sex-biased genes (SBGs) and GWAS Catalog risk-gene sets for psoriasis and atopic dermatitis; Table S5: Excel file containing whole-skin sex-associated differentially expressed genes; Table S6: Excel file containing sex-associated differentially expressed genes identified in major cell types.

## Abbreviations

The following abbreviations are used in this manuscript:

F-SBGs	Female-upregulated SBGs
M-SBGs	Male-upregulated SBGs
SBGs	Sex-biased genes
scRNA-seq	single-cell RNA-sequencing
Tregs	regulatory T cells
